# Regressions of Breast Carcinoma Syngraft Following Treatment with Piperine in Combination with Thymoquinone

**DOI:** 10.3390/scipharm85030027

**Published:** 2017-07-03

**Authors:** Wamidh H. Talib

**Affiliations:** Department of Clinical Pharmacy and Therapeutics, Applied Science Private University, Amman 11931-166, Jordan; w_talib@asu.edu.jo; Tel.: +962-6560999 (ext. 1141); Fax: +962-65232899

**Keywords:** anticancer, natural products, *Nigella sativa*, combination therapy, breast cancer

## Abstract

Thymoquinone (TQ) and piperine, the active ingredients in cumin (*Nigella sativa*) and black pepper (*Piper longum*), respectively, exhibit various bioactivities including anticancer effects. The aim of the present study is to investigate the antineoplastic activity of a combination of TQ and piperine against breast cancer implanted in mice. The antiproliferative effects of TQ, piperine, and a combination of both agents were tested against mouse epithelial breast cancer cell line (EMT6/P) using MTT assay. The isobolographic method was used to calculate the combination index (CI). Degree of angiogenesis inhibition was detected by measuring vascular endothelial growth factor (VEGF) levels in tissue culture for all treatments. EMT6/P cells were inoculated in Balb/C mice and the antitumor effect of TQ, piperine, and their combination was assessed. Changes in tumor size were calculated for all treatments. Tumor histology was examined using the hematoxylin/eosin staining protocol. Terminal deoxynucleotidyl transferase (TdT) dUTP Nick-End Labeling (TUNEL) colorimetric assay and caspase-3 activity assays were used to detect apoptosis. Serum levels of interferon (INF)-γ, interleukin (IL)-4, IL-2, and IL-10 were measured using ELISA and treatment toxicity was evaluated by measuring serum levels of aspartate transaminase (AST), alanine transaminase (ALT), and creatinine. A clear synergistic antiproliferative interaction between TQ and piperine was observed with CI value of 0.788. The combination therapy resulted in significant reduction in tumor size with percentage cure of 60% and percentage death of 0%. High degrees of apoptosis and geographical necrosis were induced in tumors treated with the combination therapy. Combination therapy caused significant decrease in VEGF expression and increased serum INF-γ levels. Normal serum levels of AST, ALT, and creatinine were observed in tumor-bearing mice treated with the combination therapy. The combination of TQ and piperine acts synergistically to target breast cancer in vitro and in vivo. This novel combination exerts its effect by angiogenesis inhibition, apoptosis induction, and shifting the immune response toward T helper1 response. This combination therapy deserves further investigation (including measurement of hypoxia-inducible factor (HIF)1α to be used in clinical studies.

## 1. Introduction

Cancer represents one of the main causes of mortality worldwide with an estimation of increase to 19.3 million new cases per year for 2025. Although cancer is a global disease, more than 50% of reported cancer mortality occurs in countries with low and middle incomes [1]. One of the highly incident cancers is breast cancer, which is the most common invasive malignancy in women [2] and is characterized by high proliferation rate, resistance to apoptosis, and unregulated differentiation [3]. Current breast cancer therapies include chemotherapy, radiation, surgery, hormonal therapy, and immunotherapy [4]. Serious side effects were reported in patients treated with conventional anticancer therapies. These side effects include bone loss, sexual dysfunction, anemia, weight loss, and menopausal symptoms [5]. 

Recent studies showed that combinations of compounds with low toxicity can provide efficient anticancer therapy by targeting many pathways essential for cancer genesis and metastasis. Chemicals from plants and foods represent attractive options for such combinations because they are less toxic and more cost effective when compared with synthetic chemicals [6]. 

Thymoquinone (TQ) is the active ingredient of *Nigella sativa*. It has various biological activities including anti-oxidant, anti-diabetic, anti-allergic, and antitumor effects [7]. In its anticancer effect, TQ acts solely or in combination with other agents to induce apoptosis in targeted cancer cells [8,9]. Piperine is an active constituent of *Piper nigrum* L. and *Piper longum* and exhibits various activities including anti-inflammatory, immunomodulatory, anti-tumor, anti-metastatic and hepatoprotective activities [10]. Inhibitions of metastasis and tissue invasion were reported as modes of action for piperine against different cancers [11,12]. Additionally, piperine can induce apoptosis in p53-dependent pathway [13]. 

The poor outcome of conventional anticancer therapies against breast cancer made combination therapies a necessary option. Thymoquinone was successfully combined with other agents to improve its therapeutic efficiency against different cancers [7,9,14]. However, its anticancer activity was not tested in combination with piperine. Therefore, in this study, we employed a combination anticancer therapy consisting of piperine and TQ to treat breast cancer in mice. We hypothesized that piperin and TQ may work synergistically by integrating different mechanisms of action to target breast cancer implanted in mice. 

## 2. Materials and Methods

### 2.1. Mice

Standard ethical guidelines were followed in this study and all experimental procedures were approved by the Research and Ethical Committee of Applied Science University (Approval Number: 2015-PHA-05). The study was conducted on 40 Balb/C female mice (4–6 weeks old, weight 21–25 g/mouse). Mice were kept in separate cages using wood shavings as bedding. The animal house conditions were: temperature around 25 °C, 50–60% humidity, alternating 12-h light/dark cycles and continuous air ventilation.

### 2.2. Chemicals, Cell Line and Culture Conditions

Thymoquinone and piperine were provided from Sigma, (St. Louis, MO, USA). EMT6/P (ECACC 96042344) mouse mammary cell line was purchased from the European Collection of Cell Cultures. Cancer cells were cultured using minimum essential medium (MEM) supplemented with 10% fetal calf serum, 1% l-glutamine, 0.1% gentamycin, and 1% penicillin-streptomycin solution. Cells were incubated at 37 °C in 5% CO_2_ and 95% humidity.

### 2.3. MTT Cell Viability Assay

Freshly growing cancer cells were harvested, washed and suspended in tissue culture media. Viability of cells was determined using trypane blue stain. Cells were dispensed (100 μL/well) into 96-well tissue culture flat bottom plates at an optimized concentration of 13,000 cells/well in a complete medium. After overnight incubation, cells were treated in triplicates with increasing concentrations of TQ (10–800 μM), piperine (50–1200 μM), and with different combinations of TQ and piperine resulting in a final volume of 200 μL/well. Treated cells were incubated for 48 h and cell viability was measured using 3-(4,5-dimethylthiazol-2-yl)-2,5-diphenyltetrazolium bromide (MTT) assay. In this assay, 100 μL of culture media were removed from each well and replaced with 10 μL of thiazolyl blue tetrazolium solution (Sigma) followed by incubation at 37 °C for 3 h. MTT solubilization solution (Sigma) was added to each well (100 μL/well), mixed and incubated for another hour. Absorbance was measured at 595 nm by microplate reader (Biotek, Winooski, VT, USA). Percentage cell viability was determined for all groups compared to untreated sample. Untreated cells were used as a negative control and cells treated with vincristine sulfate were used as a positive control.

### 2.4. Calculation of Combination Index and Data Analysis

The isobolographic method was used to measure the type of interaction between TQ and piperine. The combination index (CI) was calculated for combinations of TQ and piperine against EMT6/P cells and results were interpreted as described below [15]:

CI = (*D*)1/(*Dx*)1 + (*D*)2/(*Dx*)2 + α(*D*)1(*D*)2/(*Dx*)1(*Dx*)2

where (*Dx*)1 = IC_50_ of drug 1 (TQ) alone; (*D*)1 = IC_50_ of drug 1 (TQ) in combination with drug 2 (piperine); (*Dx*)2 = IC_50_ of drug 2 (piperine) alone (*D*)2 = IC_50_ of drug 2 (piperine) in combination with drug 1 (TQ); α = 0 for mutually exclusive or 1 for mutually nonexclusive modes of drug action.

Interpretation of results: antagonism for CI value above 1.3; moderate antagonism for CI value of 1.1 to 1.3; additive interaction for CI value of 0.9 to 1.1; slight synergism for CI value of 0.8 to 0.9; moderate synergism for CI value of 0.4 to 0.6; and strong synergism for CI value of 0.2 to 0.4.

### 2.5. Anticancer Therapy on Experimental Animals

Actively dividing EMT6/P cells were trypsinized, centrifuged, washed, counted, and suspended in complete minimum essential medium (MEM) at a density of 1 × 10^6^/mL. Trypan blue exclusion method was used to measure cell viability and a tumorigenic dose of 100,000 cells (suspended in 0.1 mL) was injected subcutaneously in the abdominal area of each mouse. Cancer cells were allowed to grow for 14 days, and a digital caliper was used to measure induced tumors. Tumor volume was calculated using the formula (A × B^2^ × 0.5), where A was the length of the longest aspect of the tumor and B was the length of the aspect perpendicular to A [16]. Tumor bearing mice were then randomly divided into four groups with closely matched tumor volumes for all groups. The following groups were studied: Group I: Control group (10 mice) were injected IP with vehicle (phosphate buffered saline) 0.1 mL daily. Group II: TQ (10 mice) were injected IP with 10 mg/kg/day of TQ. Group III: piperine (10 mice) were injected IP with 25 mg/kg/day peperine. Group IV: Combination (10 mice) were injected IP with 10 mg/kg/day TQ + 25 mg/kg/day peperine. Mice were treated for 14 days, then tumors were measured again at the end of the treatment, after which mice were sacrificed, tumors extracted, weighed and stored in 10% formalin.

### 2.6. Histological Examination of Tumor Sections

Standard hematoxylin and eosin staining procedure was used to stain dehydrated paraffin sections prepared from tumors of different treatments. Stained slides were examined using light microscope (Zeiss, Jena, Germany) equipped with a computer-controlled digital camera (Canon, Taichung, Taiwan).

### 2.7. Measuring Vascular Endothelial Growth Factor Expression in EMT6/P Cells

The effect of different treatments on vascular endothelial growth factor (VEGF) expression was measured using mouse VEGF ELISA kit (Sigma). EMT6/P cells were cultured at a concentration of (1.5 × 10^5^ cell/mL) and treated for 48 with one of the following treatments: 425 μM piperine, 80 μM TQ, combination of 425 μM piperine + 80 μM TQ and negative control which only contain MEM. Cells were processed according to the kit instructions. Briefly, cells were harvested, washed, and lysed using cell lysis buffer. Supernatants were collected and 100 μL was added to each well in the 96-well microplates coated with VEGF capture antibody, then incubated for 2.5 h. After washing, 100 μL of biotinylated detection antibody was added and incubated for 1 h followed by washing and adding 100 μL of horseradish peroxidase (HRP)-conjugated streptavidin with 45 min incubation. For color development 100 μL of 3,3′,5,5′-tetramethylbenzidine (TMB) substrate solution was added and incubated for 30 min in a dark place. Color intensity was measured at 450 nm after adding 50 μL of stopping solution.

### 2.8. DeadEnd TUNEL Colorimetric Assay to Detect Apoptosis

The Terminal deoxynucleotidyl transferase (TdT) dUTP Nick-End Labeling (TUNEL) Colorimetric Apoptosis Detection System (Promega, Fitchburg, WI, USA) was used to detect the degree of apoptosis induced by different treatments. Paraffin-embedded tumor sections of different treatments were stained as described in the kit instructions. Briefly, sections were de-paraffinized followed by rehydration then fixation using 10% buffered formalin. Proteinase K solution (20 μg/mL) was added to each slide followed by re-fixation. Sections were equilibrated by using equilibration buffer for 5–10 min at room temperature and DNA fragments were labeled by adding recombinant terminal deoxynucleotidyl transferase (rTdT) reaction mixture at 37 °C in a humidified chamber. The reaction was terminated using 2× SSC termination solvent provided in the kit. Color development was performed by adding horseradish peroxidase-labeled streptavidin followed by incubation with 3,3′-Diaminobenzidine (DAB) for 20 min in the dark. Finally, slides were mounted with glycerol and examined under the light microscope.

### 2.9. Induction of Caspase-3 Activity in EMT6/P Cells

After treatment with 80 μM TQ, 425 μM Piperine and combination (80 μM TQ + 425 μM Piperine), cells (1 × 10^6^/mL) were washed with ice-cold PBS and lysed using cell lysis buffer (caspase-3 assay kit, catalogue # ab39401; Abcam, Cambridge, MA, USA). Samples were incubated on ice for 10 min and centrifuged in microcentrifuge at 12,000× *g* for 5 min at 4 °C to precipitate the cellular debris. The caspase-3 activity in the supernatant was measured by spectrophotometry using DEVD-*p*-nitroanilide as a substrate at 405 nm and according to the manufacturer's instructions provided with the assay kit. Caspase-3 activity was measured at different time intervals (0, 6, 12, 24, 48 h).

### 2.10. Detection of IFN-γ, IL-2, IL-4 and IL-10 Serum Levels

The effect of each treatment on the immune response of tumor-bearing mice was determined using Mouse Th1/Th2 ELISA kit (Thermo Fisher Scientific, Toronto, Canada). Blood samples were collected from mice subjected to different treatments and serum samples were prepared. Interferon (IFN)-γ, interleukin (IL)-2, IL-4 and IL-10 were detected using kit instructions.

### 2.11. Determination of Aspartate Transaminase (AST) alanine Transaminase (ALT), and Creatinine Serum Levels

Assessment of liver and kidney functions in treated mice was conducted using instructions from commercially available kits (BioSystems, Barcelona, Spain).

### 2.12. Statistical Analysis

Data are presented using mean ± standard error from three independent experiments. The statistical significance among the groups was determined by using one-way analysis of variance. A *p* < 0.05 was considered significant. The IC_50_ values obtained with the different concentrations of TQ and/or piperine were calculated using nonlinear regression in Statistical Package for the Social Sciences (SPSS Inc. Released 2009. PASW Statistics for Windows, Version 18.0. Chicago, IL, USA).

## 3. Results

### 3.1. Cytotoxic Effect of Piperine and/or Thymoquinone on Mouse Breast Cancer Cells

A dose-dependent inhibition of EMT6/P cells proliferation was observed after treatment with TQ (10–800 μM) or piperine (50–1200 μM) with IC_50_ values of 390 and 870 μM, respectively (Table 1). Testing different combinations of piperine and TQ showed a clear synergistic interaction (CI = 0.788) with reduction in the IC_50_ values for both agents to 425 and 80 μM, respectively (Table 1).

### 3.2. Inhibition of Vascular Endothelial Growth Factor Expression by Different Treatments

The ability of different treatments to inhibit angiogenesis was evaluated by measuring the reduction of VEGF expression in cell culture. High levels (890.4 pg/mL) of VEGF were observed in the negative control group. Both piperine and TQ caused significant (*p* < 0.05) reduction in VEGF expression with VEGF levels of 177.5 and 632.7 pg/mL, respectively. However, the most potent inhibition was reported in the combination therapy group with VEGF levels of 84.9 pg/mL (Table 2).

### 3.3. Antitumor Effect of Thymoquinone, Piperine, and Their Combination against Breast Cancer Implanted in Mice

Significant (*p* < 0.05) reduction in tumor size was observed in mice treated with 25 mg/kg/day piperine with percentage change in tumor size of (−15.05%) compared with the negative control group, which exhibited an increase in tumor size of (78.93%). Treatment of tumor-bearing mice with 10 mg/kg/day of TQ resulted in greater reduction in tumor size with percentage change in tumor size of (−26.93%). However, the highest reduction in tumor size was observed in the combination therapy group with percentage change in tumor size of (−47.84%) (Table 3). Additionally, the highest tumor regression percent (60%) was observed in the combination therapy group, while lower percentages were observed in the groups treated with TQ and piperine with percentage regression of 30 and 10%, respectively (Table 3). Although piperine treatment caused a significant (*p* < 0.05) reduction in tumor size, this treatment failed to reduce percentage death observed in the negative control group (20%). Treatment of tumor-bearing mice with TQ resulted in a reduction in death percentage to 10%. This reduction was more obvious in the combination therapy group, where no death was reported (Table 3). 

### 3.4. Induction of Apoptosis in Tumor Sections

**Figure 1:**
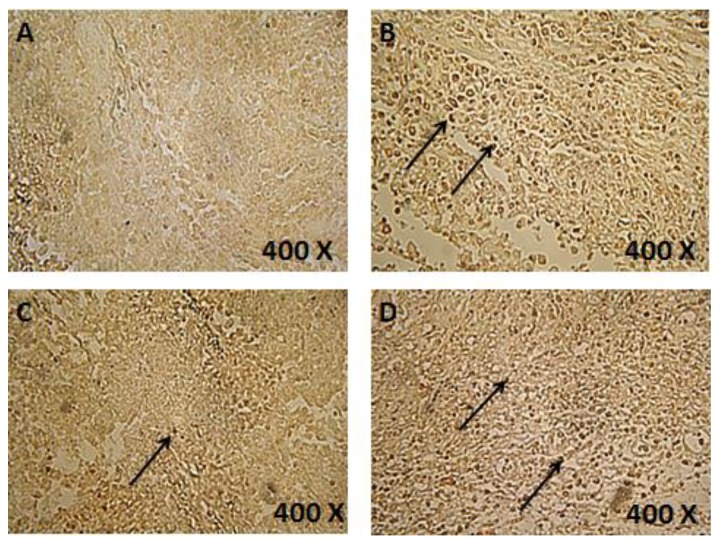
Colorimetric terminal deoxynucleotidyl transferase (TdT) dUTP Nick-End Labeling (TUNEL) assay for detection of apoptosis in tumor sections treated with vehicle (**A**) thymoquinone (TQ) 10 mg/kg (**B**) piperine 25 mg/kg (**C**) and a combination of 10 mg/kg TQ and 25 mg/kg piperine (**D**). Brown stained nuclei (arrows) indicate DNA fragmentation and nuclear condensation. Tumors of four mice for each treatment were examined to detect apoptosis.

**Figure 2:**
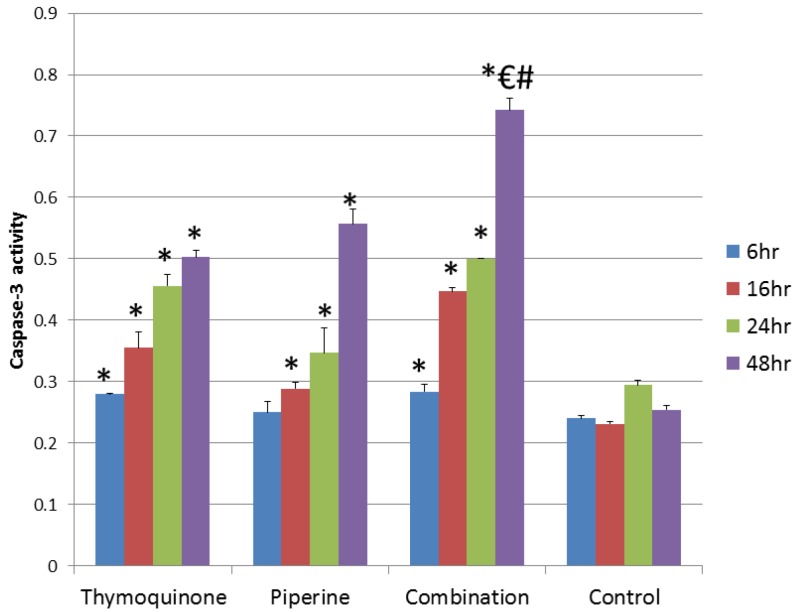
Activity of caspase-3 in EMT6/P cells after treatment with 80 μM TQ, 425 μM Piperine, and combination (80 μM TQ + 425 μM Piperine). Results are expressed as the mean optical density (405 nm) ± standard deviation (SD) (*n* = 3). * *p* < 0.05 compares the treated cell with control cell; compared with single 48 h single TQ treatment group, ^#^ *p* < 0.05; compared with 48 h single piperine treatment group ^€^ *p* < 0.05.

TUNEL colorimetric assay was used to detect apoptotic cells in tumor sections after different treatments. A limited number of apoptotic cells was observed in the negative control group. A slight increase in the number of apoptotic cells was reported in the tumor sections treated with piperine. On the other hand, TQ and combination therapy induced the highest degree of apoptosis in tumor sections (Figure 1). Further testing revealed significant elevation in the activity of caspase-3 in cells treated with TQ (80 μM), piperine (425 μM), and combination (80 μM TQ + 425 μM Piperine). The increase in caspase-3 activity was time dependent and the highest activity was observed after 48 h. The combination therapy caused the highest activation of caspase-3 in all time intervals (Figure 2). 

### 3.5. Histological Examination of Tumor Sections

**Figure 3:**
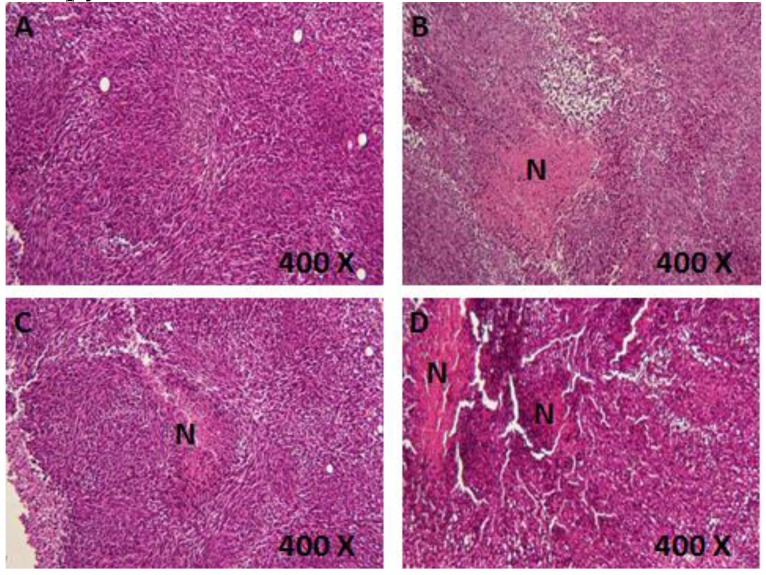
Hematoxylin and eosin staining of tumors treated with vehicle (**A**) thymoquinone (TQ) 10 mg/kg (**B**) piperine 25 mg/kg (**C**) and a combination of 10 mg/kg TQ and 25 mg/kg piperine (**D**). N: Necrotic area. Four mice were examined for each treatment.

In order to provide more details about the mechanisms of action of different treatments, tumor sections were stained using standard hematoxylin and eosin procedure and were examined to detect necrotic areas. Small necrotic regions (red areas) were observed in tumor sections treated with TQ or piperine. Combination therapy induced extensive necrosis in tumor sections (Figure 3).

### 3.6. Effect of Piperine and/or Thymoquinone on the Immune System

Mouse Th1/Th2 ELISA kit was used to measure the effect of different treatments on the production of IFN-γ, IL-2, IL-4 and IL-10. The highest levels of IFN-γ (140.1 pg/mL) were detected in the combination therapy group. Other treatments caused a slight increase in IFN-γ levels compared with the control (29.8 pg/mL) with values of 45.8 and 68.2 pg/mL for TQ and piperine, respectively. Similar results were observed for IL-2 with the highest levels observed in combination therapy. Values of other cytokines (IL-4 and IL-10) were close to each other in all treatments (Table 4).

### 3.7. Effect of Different Treatments on Serum Levels of AST, ALT, and Creatinine

In order to evaluate the effect of different treatments on liver and kidney functions, serum levels of AST, ALT, and creatinine were measured. All treatments caused no liver or kidney toxicity as indicated by low serum levels of liver enzymes and creatinine (Table 5).

## 4. Discussion

In this study, we evaluated the anticancer effect of a new combination consisting of thymoquinone (TQ) and piperine. Both agents are natural products and are proven to have anticancer effect against different cancers, including breast cancer [17,18,19,20,21]. However, the anticancer activity of their combination was not tested before. We demonstrated here that TQ and piperine can act synergistically to inhibit breast cancer in vitro and in vivo. The anticancer activity of this combination is mediated by apoptosis induction, angiogenesis inhibition, and modulation of the immune system. Our in vitro cytotoxicity results showed a dose-dependent inhibition of EMT6/P cells by TQ and piperine. Piperine exhibits cytotoxicity at higher concentration (IC_50_ = 870 μM) than TQ (IC_50_ = 390). Combination of both agents caused a reduction in IC_50_ values with clear synergistic effect. These results are consistent with the previous studies that reported a synergistic effect of TQ with different agents, including topotecan against colorectal cancer [18], docetaxel against prostate cancer [22], and diosgenin on squamous cell carcinoma [23]. In addition, combination of piperine with curcumin inhibited the development of hepatocellular carcinoma in rats [24] and increased the efficiency of docetaxel against prostate cancer [25].

Targeting angiogenesis is an important mechanism in cancer therapy as it reduces cancer cell proliferation by depriving the tumor of oxygen and nutrients [26]. In our study, both TQ and piperine worked synergistically to inhibit angiogenesis by lowering VEGF levels. These results agree with the recent findings that showed the ability of TQ and piperine to inhibit angiogenesis [27,28]. However, angiogenesis inhibition as a single target cannot cause complete tumor regression [29]. Accordingly, and in order to have a better understanding of the obtained results, we tried to explore other anticancer targets of this combination.

One of the main mechanisms used by cancer cells to survive is the inactivation of apoptosis. Thus, apoptosis induction is considered as one of the effective mechanisms to improve anticancer therapies [30]. A high degree of apoptosis was observed in tumors treated with TQ or combination therapy. On the other hand, tumors treated with piperine exhibited limited number of apoptotic cells. This result indicates that the main inducer of apoptosis in our combination is TQ. Our findings are consistent with other studies that reported the ability of TQ to induce apoptosis in different cancers including renal [31] and breast [32] cancers.

In order to have a better insight into other anticancer mechanisms induced by this combination, we measured serum levels of IFN-γ, IL-2, IL-4 and IL-10. IFN-γ and IL-2 are signature cytokines of Th1 immune response and IL-4 is the main cytokine produced in Th2 immune response. Balanced ratios of Th1/Th2 cytokines are present in healthy immune systems. On the other hand, high concentrations of Th2 cytokines were observed in patients with different types of cancer [33]. Combination of TQ and piperine induced higher concentrations of IFN-γ and IL-2 compared with single therapies. This result indicates that the combination therapy inhibits cancer cells by stimulating Th1 anticancer immune response. Such a shift in the immune response toward Th1 was reported for TQ [34] and piperine [35]. It seems that the observed anticancer Th1 immune response is a result of the combined effect of TQ and piperine.

Results obtained in the in vitro experiments were supported by in vivo results. Significant reduction (*p* < 0.05) in tumor size was observed in the group treated with the combination therapy. This combination also caused tumor regression in 60% of treated mice. Additionally, the combination therapy induced extensive necrosis in tumors and reduced the death rate to 0% compared with 20% observed in the negative control group. The high therapeutic efficiency of this combination is a direct result of activation of different anticancer mechanisms including angiogenesis inhibition, apoptosis induction, and shifting the immune response toward Th1 anticancer immune response. 

Toxicity study revealed a low level of hepatic and nephro-toxicity of the combination therapy as indicated by normal levels of AST, ALT, and creatinine. Such low toxicity was expected as we used a safe concentration of TQ (10 mg/kg), which is 10 times lower than its LD_50_ value (104.7 mg/kg) reported in mice [36] and a relatively low concentration of piperine (25 mg/kg), which is also lower than its reported LD_50_ (60 mg/kg) in female mice [37].

## 5. Conclusions

A combination of TQ and piperine can work synergistically to inhibit breast cancer in vitro and in vivo. The combination acts mainly by apoptosis induction, inhibition of angiogenesis, and shifting the immune response toward Th1 anticancer response. TQ is the main inducer of caspase-dependent apoptosis in this combination. Both agents work in angiogenesis inhibition and immune system stimulation. Further testing (including measurement of hypoxia-inducible factor (HIF)1α levels) is needed to understand the exact mechanism of action of this combination. 

## Figures and Tables

**Table 1:** The IC_50_ values and combination index (CI) for thymoquinone (TQ) and piperine against EMT6/P cell line.

Piperine IC_50_	Thymoquinone (TQ) IC_50_	Piperine IC_50_ in Combination	TQ IC_50_ in Combination	Combination Index (CI)	Interpretation
870 ± 5.02	390 ± 3.16	425 ± 5.11	80 ± 7.11	0.788	Synergism

**Table 2:** Effect of different treatments on the vascular endothelial growth factor (VEGF) expression by EMT6/P cell line.

Treatment	VEGF (pg/mL)
Negative control	890.4 ± 1.50
Thymoquinone (TQ)	632.7 ± 2.50 *
Piperine	177.5 ± 1. 90 **
Combination (80 μM TQ + 425 μM Piperine)	84.9 ± 0.97 **

Compared with the negative control group, * *p* < 0.05; ** *p* < 0.01.

**Table 3:** Effect of Thymoquinone, piperine and their combination on tumor size, cure percentage, and survival rates.

Treatment	Initial Tumor Size	Final Tumor Size	% Change in Tumor Size	% of Cured Mice	% Death
Control	275.19 ± 28.4	492.41 ± 47.9	78.93	10%	20%
Piperine	186.99 ± 31.9	158.85 ± 54.3	−15.05 *	10%	20%
Thymoquinone	151.39 ± 30.6	110.62 ± 9.1	−26.93 *	30%	10%
Combination	145.04 ± 36.5	75.64 ± 19.2	−47.84 *^,#,€^	60%	0

Data were expressed as the mean ± standard error of mean (SEM). Compared with the negative control group, * *p* < 0.05; compared with single TQ treatment group, ^#^ *p* < 0.05; compared with single Piperine treatment group ^€^ *p* < 0.05. Mice were considered cured if they had undetectable tumors in the inoculation site after 14 d treatment. Death percentage was calculated by counting the number of dead animals in each group during 14 d treatment time.

**Table 4:** Serum levels of interferon (INF)-γ, interleukin (IL)-2, IL-4, and IL-10 (pg/mL ± SEM) for different treatments.

Treatment	INF-γ	IL-2	IL-4	IL-10
Control	29.8 ± 0.08	65.2 ± 0.06	61.1 ± 0.06	60 ± 0.04
Thymoquinone (TQ)	45.8 ± 0.02 *	85 ± 0.02 *	56.2 ± 0.02 *	51.1 ± 0.05 *
Piperine	68.2 ± 0. 18 *	108.8 ± 0.08 *	44.8 ± 0.04 *	52.1 ± 0.52 *
Combination	140.1 ± 0.02 *^,#,€^	158.1 ± 0.03 *^,#,€^	55.1 ± 0.04 *^,€^	54.2 ± 0.25 *

Compared with the negative control group, * *p* < 0.05; compared with single TQ treatment group ^#^ *p* < 0.05; compared with single Piperine treatment group, ^€^ *p* < 0.05.

**Table 5:** Serum levels of AST, ALT, and creatinine after treatment with Thymoquinone (TQ), piperine and their combination.

Treatment	ALT (IU/L) ± SEM	AST (IU/L) ± SEM	Creatinine (µmol/L) ± SEM
Thymoquinone (TQ)	63.63 ± 0.025 *	24.2 ± 1.84 *	36.46 ± 1. 18 *
Piperine	46.17 ± 0.018 *	18.42 ± 2.62 *	31.82 ± 2.23 *
Combination	40.82 ± 0.007 *^,#,€^	21.15 ± 0.24 *^,#^	31.76 ± 0.08 *^,#^
Control	75.27 ± 0.029	48.15 ± 2.82	57.17 ± 1.63

Compared with the negative control group, * *p* < 0.05; compared with single TQ treatment group ^#^ *p* < 0.05; compared with single Piperine treatment group, ^€^ *p* < 0.05. ALT: alanine transaminase; AST: aspartate transaminase.
